# Gall-Bladder Agenesis and Associated Anomalies

**DOI:** 10.1155/1995/90957

**Published:** 1995

**Authors:** Constantinos Yiangou, Brian Shorey, Allan D. Spigelman

**Affiliations:** 1 Registrar Department of Surgery Central Middlesex Hospital London NW10 UK; 2 Consultant Surgeon Hillingdon Hospital Uxbridge Middlesex UK; 3 Senior Lecturer and Honorary Consultant Surgeon Academic Surgical Unit St. Mary's Hospital Medical School London W2 UK

## Abstract

Congenital absence of the gall-bladder is a rare condition. It is sometimes associated with other congenital defects. We report here two cases of gall-bladder agenesis discovered at laparoscopy. Both had a history of skeletal and cardiovascular anomalies. The investigation of patients with absent gall-bladder can be very difficult. Ultrasound scanning is usually inconclusive and further noninvasive tests should be performed to establish the diagnosis and prevent an unnecessary operation. The presence of other congenital defects should alert the clinician to the possibility of gall-bladder agenesis.


*HPB Surgery*, 1995, Vol. 8, pp. 253–255
Reprints available directly from the publisher
Photocopying permitted by license only

© 1995 OPA (Overseas Publishers Association)
Amsterdam B.V. Published in The Netherlands
by Harwood Academic Publishers GmbH
Printed in Malaysia

### Gall-Bladder Agenesis and Associated Anomalies

CONSTANTINOS YIANGOU*

Registrar, Department of Surgery, Central Middlesex Hospital, London NW10

BRIAN SHOREY

Consultant Surgeon, Hillingdon Hospital, Uxbridge, Middlesex

ALLAN D. SPIGELMAN**

Senior Lecturer and Honorary Consultant Surgeon, Academic Surgical Unit,
St. Mary's Hospital Medical School, London W2

*(Received April 1, 1994)*

Congenital absence of the gall-bladder is a rare condition. It is sometimes associated with other congenital defects. We report here two cases of gall-bladder agenesis discovered at laparoscopy. Both had a history of skeletal and cardiovascular anomalies. The investigation of patients with absent gall-bladder can be very difficult. Ultrasound scanning is usually inconclusive and further noninvasive tests should be performed to establish the diagnosis and prevent an unnecessary operation. The presence of other congenital defects should alert the clinician to the possibility of gall-bladder agenesis.

KEY WORDS: Gall bladder agenesis congenital anomalies investigation of

### INTRODUCTION

Congenital absence of the gall-bladder is a rare condition. It is sometimes associated with cardiac defects, polycystic kidneys, gastrointestinal and other abnormalities (eg, cleft lip and palate) [1–3].

We report two cases of gall-bladder agenesis associated with cardiac defects and skeletal abnormalities. The particular skeletal defects found in the present cases have not been reported before.

### *Case 1*

A 42 year old woman presented to the Surgical Outpatients Department with a 4 month history of intermittent right hypochondrial pain, provoked by fatty food. As a child, she underwent pyloromyotomy for congenital hypertrophic pyloric stenosis and also ligation of a patent ductus arteriosus. Other congenital abnormalities included absence of the right thumb and spina bifida occulta. Preoperative liver function tests were within normal limits. An ultrasound scan of her biliary tree did not identify the gall-bladder. An oral cholecystogram was then performed, during which the gall-bladder did not opacify. This was thought to be consistent with a shrunken gall-bladder secondary to chronic cholecystitis.

Based on the above tests, the decision was made to perform cholecystectomy. At laparoscopy, no gall-bladder was found in the gall-bladder fossa. The

*Address for correspondence*:
*Present Appointment: Research Fellow, Department of Oncology, Charing Cross Hospital, London. **To whom correspondence and requests for reprints should be addressed, at St. Mary's Hospital Medical School, London W2.

extrahepatic biliary tree was dissected out and neither the gall-bladder nor the cystic duct were identified. A diagnosis of congenital absence of the gall-bladder was therefore made. After an uneventful postoperative recovery an upper gastrointestinal endoscopy showed a small hiatus hernia with no evidence of reflux oesophagitis. Six months after the operation she remains well and, apart from two episodes of upper abdominal discomfort, is asymptomatic.

*Case 2*

A 27 year old woman with a family history of gallstones (maternal grandmother and aunt) presented with a 2 year history of intermittent right upper quadrant abdominal pain which was worse after fatty meals. As a child, she underwent surgery to close an atrial septal defect and to correct a lower limb deformity secondary to an absent fibula. An ultrasound scan of her upper abdomen was thought to demonstrate a contracted gall-bladder. Oral cholecystography was then performed, during which the gall-bladder failed to opacify.

The diagnosis of chronic cholecystitis secondary to cholelithiasis was made and an elective laparoscopic cholecystectomy planned. At laparoscopy no gall-bladder was found. Due to subhepatic adhesions, laparoscopic dissection of the extrahepatic biliary tree was difficult. A laparotomy was, therefore,performed and a careful exploration confirmed the absence of the gall-bladder as did a peroperative cholangiogram. Her postoperative recovery was good. However, she still gets intermittent right hypochondrial pain and further investigations of the upper gastrointestinal tract are planned.

### DISCUSSION

The gall-bladder develops from the hepatic diverticulum, which arises from the distal foregut,in the third to the fourth week of intrauterine life[4]. Agenesis of the gall-bladder was first described in 1701[5]. It has an estimated incidence of 0.01-0.05% in patients undergoing a laparotomy for suspected biliary disease[6,7] and 0.04% at post-mortem[8].

Absence of the gall-bladder can occur as part of a syndrome and the congenital anomalies described include, polycystic kidneys, cardiopulmonary defects, skeletal deformities, clift lip and cleft palate [1,2]. It was also shown to be associated with thalidomide anomalies[9] and other gastrointestinal defects which include: duodenal stenosis, pylorospasm, imperforate anus and absence of the appendix[3]. One of our patients had pyloric stenosis as an infant and both patients had cardiac and skeletal defects.

In a previous study [1], there were four children in the multiple anomaly group,they all died of their non-biliary defects which were similar to the ones mentioned above, but of greater severity. The skeletal abnormalities included malformation of the left hand, forearm, fifth digit and a prominent occiput. The skeletal defects of our two patients (absence of the thumb and fibula) have not been reported before.

We suggest that patients suspected of having symptomatic gall stones usually undergo abdominal ultrasound scanning in the first instance. If this fails to show the gall-bladder, then an oral cholecystogram should be done. Non-opacification of the gall-bladder at oral cholecystography implies that the gall-bladder is either nonfunctioning or absent.

Further non-invasive tests may resolve the situation. An abdominal CT scan should be undertaken, and if the gall bladder is still not seen endoscopic retrograde cholangiopancreatography (ERCP) should follow. This will almost certainly exclude an abnormally sited gall-bladder and may demonstrate a vestigial cystic duct[10]. If all the above investigations are inconclusive, then a laparoscopy should be performed to look for a shrunken gall bladder or other pathology that the previous tests might have missed.

The two cases reported here, indicate that the presence of other congenital abnormalities, especially skeletal defects, should alert the clinician to the possibility of gall-bladder agenesis.

### REFERENCES

1. Bennion, R.S., Thompson, J.E., Tompkins, R.K. (1988) Agenesis of the gall-bladder without extrahepatic biliary atresia. *Surgery*, **1260**, 1257–60.
2. Turkel, S.B., Swanson, V., Chandrasoma, P. (1983) Malformations associated with congenital absence of the gall-bladder. *J. Med. Genet.*, **20**, 445–9.
3. Smithells, R.W. (1973) Defects and disabilities of thalidomide children. *Br. Med. J.*, **1**, 269–72.
4. Gray, S.W., Skandalakis, J.E. (1972) Extrahepatic biliary ducts and the gall-bladder.In Embryology for Surgeons, pp. 229–62. London: W.B. Saunders.
5. Bower, J.O. (1928) Congenital absence of the gall-bladder. *Ann. Surg.*, **88**, 80–90.
6. Allan, S., Hurrell, T. A. (1974) Agenesis of the gall-bladder and cystic duct: a report of the three cases. *Br.J. Surg.*, **61**, 145–6.
7. Ahlberg, J., Angelin, B., Einarsson, K., Leijd, B. (1978) Biliary lipid composition and bile acid kinetics in patients with agenesis of the gall-bladder, with a note on the frequency of this anomaly. *Acta Chir. Scand.*, **482**, 15–20.
8. McIlrath, D. C., ReMine, W. H., Baggenstoss, A. H. (1962) Congenital absence of the gall-bladder and cystic duct. *JAMA*, **180**, 782–3.
9. James, N. K., Leaper, D. J. Further problems with thalidomide. *J.R. Coll. Surg. Edinb.* **34**, 167–8.
10. Hershman, M. J. Southern, S. J., Rosin, R. D. (1992) Gall-bladder agenesis diagnosed at laparoscopy. *J.R.S. Med.* **85**, 702–3.

